# Construction and application of a CRISPR/Cas9-assisted genomic editing system for *Corynebacterium glutamicum*

**DOI:** 10.1186/s13568-021-01231-7

**Published:** 2021-05-19

**Authors:** Chengzhen Yao, Xiaoqing Hu, Xiaoyuan Wang

**Affiliations:** 1 State Key Laboratory of Food Science and Technology, Jiangnan University, Wuxi, China; 2 International Joint Laboratory On Food Safety, Jiangnan University, Wuxi, Jiangsu China; 3 Key Laboratory of Industrial Biotechnology, Ministry of Education, School of Biotechnology, Jiangnan University, Wuxi, China

**Keywords:** *Corynebacterium glutamicum*, CRISPR/Cas9, Metabolic engineering, Genomic editing, l-Glutamic acid fermentation

## Abstract

*Corynebacterium glutamicum* is widely used as microbial cell factory for various bioproducts, but its genomic editing efficiency needs to be improved. In this study, a highly efficient CRISPR/Cas9-assisted genomic editing system for *C. glutamicum* was constructed. This system mainly involves a plasmid and can be used for both gene insertion and deletion in the chromosome of *C. glutamicum*. The recombinant plasmid for the target gene containing all the editing elements, and first constructed it in *E. coli*, then purified and transformed it into *C. glutamicum*. This temperature-sensitive plasmid was cured at high temperature after the genomic editing was completed in *C. glutamicum*. Using this genetic editing system, the genetic editing efficiency in *C. glutamicum* ATCC 13032 could reach 95%. The whole work of editing could be done in 8–9 days and showed most time-saving compared to the reported. Using this system, the native promoter of *gdhA1* in ATCC 13032 has been replaced with the strong promoter PtacM, and more than 10 genes in ATCC 13032 have been deleted. The results demonstrate that this CRISPR/Cas9-assisted system is highly efficient and very suitable for genome editing in *C. glutamicum*.

## Introduction

*Corynebacterium glutamicum* is an ideal industrial strain on account of high growth velocity and food-safety (Liu et al. 2016). Its traditional utilization in industries are for various amino acids production such as l-glutamic acid (Wada et al. 2016; Wen and Bao 2019), l-threonine (Lv et al. 2012; Wei et al. 2018), branched-chain amino acid (Schwentner et al. 2018; Wang 2019; Wang et al. 2019), l-lysine (Xiao et al. 2020), nonprotein amino acid (Mindt et al. 2019; Shi et al. 2018) and so on. *C. glutamicum* are one of most important model organisms in microbial cell factories due to decade studies, increasing other bioproducts metabolic pathway have been constructed in *C. glutamicum*, for example, biofuels (Sasaki et al. 2019; Zhang et al. 2019b), organic acid (Fukui et al. 2019), enzyme preparations (Yang et al. 2019) and other natural compounds (Braga et al. 2018; Cheng et al. 2019; Zha et al. 2018) and so on. The metabolic engineering of *C. glutamicum* to produce various bioproducts indicated its attractive prospect.

Metabolic engineering studies are most important part of synthetic biology for *C. glutamicum*. With the development of molecular biology, the cell factories’ establishment was inclined to rational design of metabolic pathway including Design, Construction, Evaluation, and Optimization (Chen et al. 2017). Hence, some more efficient molecular tools must be constructed and used to reestablish metabolic pathway. To date, although there are some gene editing methods of *C. glutamicum* have been reported, there were some obvious deficiencies like low efficiency and time-consuming. W. Jager et al. (Jager et al. 1992) was the first to report SacB-based genomic editing in *C. glutamicum* and Tan (Tan et al. 2012) improving this method and successfully constructed plasmid pDXW-3, which is a traceless editing method and needs two-step homologous recombination. Cre/loxP-based recombination system was first found in a plasmid of prophage of bacteriophage P1 (Austin et al. 1981) and Nobuaki Suzuki et al. (Suzuki et al. 2004) first reported the Cre/loxP-based method for editing of *C. glutamicum* genome, this method was suitable to large region (56 kb) deletion. Jinyu Hu et al. (Hu et al. 2013) constructed multiple-gene-deletion system based on Cre/loxP-mediated method in *C. glutamicum*, this method need three plasmids to edit genome: pBluescript II SK, pDTW202 and pDTW109, pBluescript II SK was used as vector of homologous arms and kanamycin cassette, pDTW202 was kanamycin cassette carrier (lox66 and lox71 sequence were added to each end of kanamycin resistance gene), temperature-sensitive plasmid pDTW109 was used to delete kanamycin cassette from targeted gene locus in chromosome. This method will leave residual sequence in the genomic loci of interest and require two electrical conversions. These methods above are often show low efficiency (< 5%) and need at least 20 days in *C. glutamicum*.

*Streptococcus pyogenes* type II CRISPR/Cas9-assisted genomic editing and CRISPRi technology are generalizable to microbiologic cell factories, such as *C. glutamicum* (Cameron Coates et al. 2019; Cleto et al. 2016; Liu et al. 2017)*, Escherichia coli* (Jiang et al. 2015; Pyne et al. 2015), *Bacillus subtilis* (Altenbuchner 2016; Westbrook et al. 2016), *Lactobacillus* (Oh and van Pijkeren 2014; Song et al. 2017), *Aspergillus* (Nodvig et al. 2018; Zhang et al. 2016), *Saccharomyces cerevisiae* (Mitsui et al. 2019; Zhang et al. 2019a). The CRISPR/Cas9 mutation and CRISPRi (Cleto et al. 2016; Gauttam et al. 2019) can fine-tune gene expression level in genome and have been used to study metabolic engineering in *C. glutamicum*, hence, some strategies for *C. glutamicum* genomic editing had been documented (Cho et al. 2017; Peng et al. 2017; Wang et al. 2018). Jiang (Jiang et al. 2017) was failed to construct CRISPR/Cas9-assisted genomic editing system, he/she speculated Cas9 protein might toxic to *C. glutamicum*, and reported *Francisella novicida* CRISPR/Cpf1-assisted genome editing system, the efficiency of codon saturation mutagenesis reached near 100% while shown low efficiency in large gene deletion (> 1 kb). Peng (Peng et al. 2017) optimized codon of Cas9 and limit its expression in *C. glutamicum* to make the cells survived in media, he used two plasmids to express Cas9 nuclease and targeted sgRNA separately. The sgRNA vector was constructed as two-step method: link targeted sgRNA (including N20 sequence) and homologous repaired arm to the vector pFST respectively, electroporation for twice to introduce vector pFSC and pFST into *C. glutamicum*, so the method was tiring and time-consuming in practical usage.

In this research, we succeed in construction of a single-plasmid CRISPR/Cas9-assisted genomic editing for *C. glutamicum*, all elements including gene *cas9*, sgRNA and homologous arms were designed in one temperature-sensitive plasmid pCCG1 or pCCG2, the whole editing work could be done in 8–9 days and 5 days for continuous editing. The editing efficiency was about 50%–95% in *C. glutamicum* ATCC 13032, this method was suitable to both small and large (> 3 kb) targeted gene mutation. Meanwhile, this strategy was extended examined in *C. glutamicum* ATCC 14067 and *C. glutamicum* ssp. *lactofermentum* (Shi et al. 2019) (a strain of *C. glutamicum* for l-isoleucine production, genomic sequence is similar to *C. glutamicum* ATCC 13869). Up to now, our strategy demonstrated high efficiency in editing procedure and time-saving compared to the reported.

## Materials and methods

### Strains, plasmids, and growth conditions

All bacterial strains and plasmids used in this study are listed in Table 1. *E. coli* DH5α was cloning host for vectors’ construction. *C. glutamicum* ATCC 13032 are mainly used strain for editing strategy, meanwhile, *C. glutamicum* ATCC 14067 and *C. glutamicum* ssp. *lactofermentum* as supplementary strains used in this study. *E. coli* DH5α were cultured in Luria–Bertani (LB) media (tryptone 10 g/L, yeast extract 5 g/L, NaCl 10 g/L, pH 7.0–7.2, LB agar supplemented with 15 g/L agar) at 37 °C. *C. glutamicum* is cultured in LBHIS media (d-sorbitol 91 g/L, tryptone 5 g/L, yeast extract 2.5 g/L, NaCl 5 g/L, brain heart infusion 1.85 g/L, pH 7.2–7.4, LBHIS agar supplemented with 15 g/L agar) at 30 °C. *C. glutamicum* harboring pCCG1/pCCG2 was cultured at 28 °C, and 37 °C for plasmid curing. Adding kanamycin 30 mg/L for *E. coli* DH5α and 20 mg/L for *C. glutamicum* as needed. The seed media (g/L): glucose·H_2_O 25, corn extract 20, KH_2_PO_4_ 1, MgSO_4_·7H_2_O 0.4, urea 5, pH 7.2–7.4. l-glutamic acid fermentation media (g/L): glucose·H_2_O 110, corn extract 1, KH_2_PO_4_ 2, MgSO_4_·7H_2_O 0.8, MnSO_4_·H_2_O 0.01, FeSO_4_·7H_2_O 0.02, pH 7.2–7.4.

**Table 1:** Bacterial strains and plasmids used in this study

Strains/plasmids	Characteristics	Source or reference
Strains		
* E. coli*		
* E. coli* DH5α	F^−^, Φ80d/*lacZ*ΔM15, Δ (*lacZYA*-*argF*)U169, *deoR*, *recA1*, *endA1*, *hsdR17*(r_k_^−^m_k_^+^), *phoA*, *supE44*, λ^−^, *thi-1*, *gyrA96*, *relA*	TaKaRa
* E. coli str. K-12 substr.* W3110	Wild strain	ND
* C. glutamicum*		
* C. glutamicum* ATCC 13032	Wild strain	ND
* C. glutamicum* ATCC 14067	Wild strain	ND
* C. glutamicum* ssp. *lactofermentum*	A strain of *C. glutamicum* for l-isoleucine production	Shi et al. (2019)
CGN001	*C. glutamicum* ATCC 13032, Δ*ldh*	This study
CGN002	*C. glutamicum* ATCC 13032, Δ*eutD*	This study
CGN003	*C. glutamicum* ATCC 13032, Δ*gabP*	This study
CGN004	*C. glutamicum* ATCC 13032, Δ*glnA1*	This study
CGN005	*C. glutamicum* ATCC 13032, Δ*glnA2*	This study
CGN006	*C. glutamicum* ATCC 13032, Δ*alaT*	This study
CGN007	*C. glutamicum* ATCC 13032, Δ*argR*	This study
CGN008	*C. glutamicum* ATCC 13032, Δ*gabTD*	This study
CGN009	*C. glutamicum* ATCC 13032, Δ*aceAB*	This study
CGN010	*C. glutamicum* ATCC 13032, Δ*poxB*	This study
CGG001	*C. glutamicum* ATCC 13032, Φ(PtacM-*gdhA1*)	This study
CGG002	CGG1, Δ*gabP*::*gadB*	This study
CGG003	*C. glutamicum* ATCC 13032, Δ*gabP*::*gadB*	This study
CGG004	CGG003, Δ*eutD*::*gdhA*	This study
CGG005	CGG004, Δ*gabTD*	This study
CGC001	*C. glutamicum* ATCC 13032, Δ*eutD*::*speC*	This study
CGY001	*C. glutamicum* ATCC 14067, Δ*gabP*	This study
CGY002	*C. glutamicum* ssp. *lactofermentum*, Δ*gabP*	This study
Plasmids		
pDTW109	Temperature-sensitive plasmid in *C. glutamicum*, Cm^r^	Hu et al. (2013)
pFSC	A plasmid harboring codon-optimized *cas9* gene, kan^r^	Peng et al. (2017)
pBluescript II SK	A plasmid containing PUC *ori* replicon, amp^r^	Hu et al. (2013)
pEC-XK99E	E. *coli*—*C. glutamicum* shuttle plasmid, kan^r^	Hu et al. (2014)
pJYW-5-*gdhA*	A recombination plasmid harboring *gdhA* under promoter PtacM, kan^r^	This study
pJYW-5-*speC*	A recombination plasmid harboring *speC* under promoter PtacM, kan^r^	This study
pJYW-5-*gadB*	A recombination plasmid harboring *gadB* under promoter PtacM, kan^r^	This study
pJYW-5-*gdhA1*	A recombination plasmid harboring *gdhA1* under promoter PtacM, kan^r^	This study
pCCG1	E. *coli*—*C. glutamicum* shuttle plasmid, CRISPR/cas9 vector, pBL1^TS^, kan^r^	This study
pCCG2	A vector derive from pCCG1, pBL1^TS^, kan^r^	This study
pBS-sgRNA	pBluescript II SK harboring sgRNA sequence	This study
pCCG1-*ldh*	pCCG1 harboring *ldh* deletion elements, kan^r^	This study
pCCG1-*eutD*	pCCG1 harboring *eutD* deletion elements, kan^r^	This study
pCCG1-*gabP*	pCCG1 harboring *gabP* deletion elements, kan^r^	This study
pCCG1-*glnA1*	pCCG1 harboring *glnA1* deletion elements, kan^r^	This study
pCCG1-*glnA2*	pCCG1 harboring *glnA2* deletion elements, kan^r^	This study
pCCG1-*alaT*	pCCG1 harboring *alaT* deletion elements, kan^r^	This study
pCCG1-*argR*	pCCG1 harboring *argR* deletion elements, kan^r^	This study
pCCG1-*gabTD*	pCCG1 harboring *gabTD* deletion elements, kan^r^	This study
pCCG1-*aceAB*	pCCG1 harboring *aceAB* deletion elements, kan^r^	This study
pCCG1-*poxB*	pCCG1 harboring *poxB* deletion elements, kan^r^	This study
pCCG1-PtacM-*gdhA1*	The plasmid to replace *gdhA1* native promoter with PtacM and artificial RBS, kan^r^	This study
pCCG1-Δ*alaT*::*gdhA*	The plasmid to insert *gdhA* into *alaT*, kan^r^	This study
pCCG2-Δ*alaT*::*gdhA*	The plasmid to insert *gdhA* into *alaT*, kan^r^	This study
pCCG1-Δ*eutD*::*speC*	The plasmid to insert *speC* into *eutD*, kan^r^	This study
pCCG1-Δ*gabP*::*gadB*	The plasmid to insert *gadB* into *gabP*, kan^r^	This study
pCCG1-Δ*gabP*::*gadB2-gadB1*^*m*^	The plasmid to insert cluster *gadB2B1*^*m*^ into *gabP*, kan^r^	This study
pCCG1-Δ*eutD*::*gdhA*	The plasmid to insert *gdhA* into *eutD*, kan^r^	This study
pCCG1-*gabP* (14067)	pCCG1 harboring *gabP* deletion elements in ATCC 14067, kan^r^	This study
pCCG1-*gabP* (ssp. *lactofermentum*)	pCCG1 harboring *gabP* deletion elements in ssp. *lactofermentum*, kan^r^	This study

ND: no data; *C. glutamicum* ATCC 13032, GeneBank GI: 58036263; *C. glutamicum* ATCC 14067, GeneBank GI: 1229082175; the genomic sequencing of *C. glutamicum ssp. lactofermentum* was completed by our research group; Cm^r^, chloramphenicol resistance gene; amp^r^, ampicillin resistance gene; kan^r^, kanamycin resistance gene; pBL1^TS^, temperature-sensitive replicon in *C. glutamicum*, derived from the plasmid pBL1 (GeneBank GI: 164604819), the replicase is active when temperature between 25 and 28 °C. Targeted gene deletion elements including N20 sequence, sgRNA and homologous repair arms

### Plasmid construction

The main primers used in this study are given in Table 2. Kanamycin resistance gene (*kan*^r^) with its native promoter was amplified from plasmid pEC-XK99E using primers P1/P2; temperature-sensitive replicon pBL1^TS^ in *C. glutamicum* was amplified from plasmid pDTW109 using primers P3/P4; PUC *ori* replicon in *E. coli* was amplified from plasmid pBluescript II SK using primers P5/P6, fused three fragments above to single Fx1 fragment with overlap PCR method, *lacI*^q^-*cas9*-T1T2 terminator fragment (Fx2) was amplified from pFSC using primers P7/P8, the fragment Fx2 contain three units: DNA-binding transcriptional repressor (*LacI*^*q*^), *cas9* gene (under inducible promoter *Ptrc*) and T1T2 terminator. Ligate *Not*I-*Apa*I-digested Fx1, *Not*I-*Sma*I-digested Fx2 and artificial synthetic PtacM promoter (annealed primers P9/P10) with T4 ligase (Thermo, USA), the products were transformed into *E. coli* DH5α to obtain plasmid pCCG-zero. Extracted the plasmid pCCG-zero from *E. coli* DH5α using TIANprep Mini Plasmid Kit (TIANGEN, China), then inserted the synthetic multiple cloning site (MCS, annealed primers P11/P12) between *Bam*HI-*Sma*I-digested pCCG-zero, the new plasmid was named as pCCG1. Amplified fragment Fx3 from plasmid pCCG1 using primers P13/P14, amplified fragment Fx4 from plasmid pEC-XK99E using primers P15/P16, amplified Fx5 fragment from plasmid pDTW109 using primers P17/18, ligate *Not*I-*Xma*JI-digested Fx3, *Xba*I-*Xho*I-digested Fx4 and *Xho*I-*Not*I-digested Fx5 using T4 ligase, the products were transformed into *E. coli* DH5α to construct a new plasmid pCCG2. Both plasmids pCCG1 and pCCG2 can utilize to editing genome of *C. glutamicum.*

**Table 2:** The main primers used in this study

Primers	Sequence
P1	CAT*GCGGCCGC*TTAGCTTGCAGTGGGCTTACATGG
P2	CGG*TCCGGA*AACCCCAGAGTCCCGCTCAG
P3	CGG*TCCGGA*GCAGTCATGTCGTGCTAATGTGTAAAAC
P4	CCG*CTCGAG*CATTGTCAACAACAAGACCCATCAT
P5	CCG*CTCGAG*GGGATAACGCAGGAAAGAACATG
P6	GC*GGGCCC*CCTTAACGTGAGTTTTCGTTCCACT
P7	CAT*GCGGCCGC*CCAGCTCATAGACCGTATCCAAAG
P8	TCC*CCCGGG*TTTCCTGCGTTATCCCCTGATT
P9	CTTGAGCTGTTGACAATTAATCATCGTGTGGTACCATACTAGTGGATCCTTCCTACCC
P10	GGGTAGGAAGGATCCACTAGTATGGTACCACACGATGATTAATTGTCAACAGCTCAAGGGCC
P11	GATCCTTCCTAGCTAGCTTGGTTGGCGCGCCAATACTTAAGCCC
P12	GGGCTTAAGTATTGGCGCGCCAACCAAGCTAGCTAGGAAG
P13	CAT*GCGGCCGC*CAGTGGAACGAAAACTCACGTTAAG
P14	GCT*CCTAGG*AGCTCATAGACCGTATCCAAAGCAT
P15	GC*TCTAGA*TAGCTTGCAGTGGGCTTACATGG
P16	ATT*CTCGAG*TCACGTAGCGATAGCGGAGTGT
P17	ATT*CTCGAG*CTGGCACGCATAGCCAAGCTAG
P18	CAT*GCGGCCGC*CATTGTCAACAACAAGACCCATCAT
P19	GC*TCTAGA*GCCTGGGGTGCCTAATGAGT
P20	CATG*CCATGG*TACAACGTCGTGACTGGGAAAAC
ldhsg-F	GT*GGATCC*CAGCATGTAGCGGAATCGAGGTTTTAGAGCTAGAAATAGCAAGTTAAAAT
ldh-U-F	CCG*CTCGAG*TGCTTCCAGACGGTTTCATC
ldh-U-R	CACCTTGCGATCATCGACATAAGGGCTCCACTTCCTACGG
ldh-D-F	CCGTAGGAAGTGGAGCCCTTATGTCGATGATCGCAAGGTG
ldh-D-R	TTCCTA*GCTAGC*ATCGGCCAAGGTCAAAGTG
eutDsg-F	CG*GGATCC*GTCGCCGTTGTGGACCATCAGTTTTAGAGCTAGAAATAGCAAGTTAAAAT
eutD-U-F	CCG*CTCGAG*ACAAATTCATGGGAGGTGCC
eutD-U-R	AAGCAAGGCAAGCACGTTGGCGCAAGAAGATGCCAGACT
eutD-D-F	AGTCTGGCATCTTCTTGCGCCAACGTGCTTGCCTTGCTT
eutD-D-R	GC*TCTAGA*GCGTAGATTCCTGCTGGACC
gabPsg-F	GT*GGATCC*GTGGAGGCATTGATCACCACGTTTTAGAGCTAGAAATAGCAAGTTAAAAT
sgRNA-R	CACGACAGGTTTCCCGACTG
gabP-F	*GTGGTACCATACTAGTGGATCC*GTGGAGGCATTGATC
gabP-U-F	CAGTCGGGAAACCTGTCGTGTGCCACTTTCACCGACTTGG
gabP-U-R	CACAAACGCCAGGGAGTACAGCAGGGATACTTCGGCGATG
gabP-D-F	CATCGCCGAAGTATCCCTGCTGTACTCCCTGGCGTTTGTG
gabP-D-R	*GCCAACCAAGCTAGCTAGGAA*CCGTGATGCTGCCTCTTCTAG
glnA1sg-F	CGT*GGATCC*CGTGGAGCATGGTGTTGAAGGTTTTAGAGCTAGAAATAGCAAGTTAAAAT
glnA1-U-F	ATT*CTCGAG*ACAATAGCAATAACCCAGGAAACAC
glnA1-U-R	AGGAGTTCAGGGTTGCGTTGCGAGGTCTGGCAGGAGATTC
glnA1-D-F	GAATCTCCTGCCAGACCTCGCAACGCAACCCTGAACTCCT
glnA1-D-R	TGC*TCTAGA*CATCTGAACTGATCGGCATCTAG
glnA2sg-F	CGT*GGATCC*GAAATATCCGCCGTTGTCAGGTTTTAGAGCTAGAAATAGCAAGTTAAAAT
glnA2sg-R	GGATTTGTTGTGGGGCTTGTCACGACAGGTTTCCCGACTG
glnA2-F	CGTGGATCCGAAATATCCGCCGTTGTCAG
glnA2-U-F	CAGTCGGGAAACCTGTCGTGACAAGCCCCACAACAAATCC
glnA2-U-R	CCAGATCCATCGACATTCCATTCGTGTTCCTACCTACCGTTTG
glnA2-D-F	CAAACGGTAGGTAGGAACACGAATGGAATGTCGATGGATCTGG
glnA2-D-R	TTCCTA*GCTAGC*CTCATCGGAGCAGGAGTAAGC
gdhA1sg-F	CGT*GGATCC*GCAAACGCCTAGGATGTACAGTTTTAGAGCTAGAAATAGCAAGTTAAAAT
gdhA1-F	*GTGGTACCATACTAGTGGATCC*GCAAACGCCTAGGATG
gdhA1-U-F	CAGTCGGGAAACCTGTCGTGTGATGCGGTAGCGGTTCCTTTG
gdhA1-U-R	GTTCACATCAACCGGCTTGTCATAC
gdhA1-D-F	GTATGACAAGCCGGTTGATGTGAACGGGGAAGAATTAGGCAGGCATC
gdhA1-D-R	*AAGCTAGACCCGGGCTTAAG*CTCACGAAGTAAACGCAGCCGTAG
alaTsg-F	CGT*GGATCC*GGTTGCCAGGCTGATGTGCTGTTTTAGAGCTAGAAATAGCAAGTTAAAAT
alaTsg-R	ATT*CTCGAG*CACGACAGGTTTCCCGACTG
alaT-U-F	ATT*CTCGAG*CGGGGTAATGCCATAACGAG
alaT-U-R	GAATAGCGTGCTGAGCTGGGCGGAATAATGCCTTTGGAGGT
alaT-D-F	ACCTCCAAAGGCATTATTCCGCCCAGCTCAGCACGCTATTC
alaT-D-R	TTCCTA*GCTAGC*GACGCAGCAAGACCTGACATAC
argRsg-F	CGT*GGATCC*TCGCGGGCGATGCTATCTACGTTTTAGAGCTAGAAATAGCAAGTTAAAAT
argRsg-R	CAAAGCCAATCATGTAGGAGTTGCACGACAGGTTTCCCGACTG
argR-F	*TGGTACCATACTAGTGGATCC*TCGCGGGCGATGCTATCTAC
argR-U-F	CAGTCGGGAAACCTGTCGTGCAACTCCTACATGATTGGCTTTG
argR-U-R	CGAGAACGAAAACGGTGTCATAGTTGTACCTGGCTGGTGACTT
argR-D-F	AAGTCACCAGCCAGGTACAACTATGACACCGTTTTCGTTCTCG
argR-D-R	*TGGCGCGCCAACCAAGCTAGC*AACCTGGTCGTTGCCCTTAC
gabsg-F	CG*GGATCC*CCAGTAATCAACCCCAGCGAGTTTTAGAGCTAGAAATAGCAAGTTAAAAT
gab-U-F	CCG*CTCGAG*ACGAGTTCGCTGATTTGGATG
gab-U-R	CAAGTCGGTGAAAGTGGCAACATGGTGAGGTTGGTCCGTC
gab-D-F	GACGGACCAACCTCACCATGTTGCCACTTTCACCGACTTG
gab-D-R	GC*TCTAGA*GGTGCCTAAAACAAAGAATCCAAG
aceABsg-F	CGT*GGATCC*GGAAATCCTCGTACGCCTCTGTTTTAGAGCTAGAAATAGCAAGTTAAAAT
aceABsg-R	GCCTCATCGGTGTCGTTGTAACACGACAGGTTTCCCGACTG
aceAB-F	*TGGTACCATACTAGTGGATCC*GGAAATCCTCGTACG
aceAB-U-F	CAGTCGGGAAACCTGTCGTGTTACAACGACACCGATGAGGC
aceAB-U-R	GTATCCGAGGATGGACTGGCAGGAACTCGGCGCAATGGGCT
aceAB-D-F	GGAACTCGGCGCAATGGGCT
aceAB-D-R	*TGGCGCGCCAACCAAGCTAGC*CCAGGGTTCGCTACGGAATC
poxBsg-F	GT*GGATCC*GGTCACCGGATACTTCACCGGTTTTAGAGCTAGAAATAGCAAGTTAAAAT
poxB-F	*GTGGTACCATACTAGTGGATCC*GGTCACCGGATACTTC
poxB-U-F	CAGTCGGGAAACCTGTCGTGGTTGCACTGCATGATCGGTT
poxB-U-R	CGCTGAAGGCTGTGGTGTTT
poxB-D-F	AAACACCACAGCCTTCAGCGTCGCAGTAACCAGAGCATTCC
poxB-D-R	*AAGCTAGACCCGGGCTTAAG*GTTTTCGAGGCGACCAGACAG
speC-F	ATT*CTCGAG*TCACATGTTCTTTCCTGCGTTATC
speC-R	*AAGCAAGGCAAGCACGTTG*TTACTTCAACACATAACCGTACAACCG
gdhA-F	*AGTCTGGCATCTTCTTGCGC*GACGTTTGAGCTGTTGACAATTAATC
gdhA-R	GAGCTCGAATTCTTAAATCACACC
gadB-F	*CATCGCCGAAGTATCCCTGC*ATTTTGGGGAAGAATTAGGCAG
gadB-R	*CGCCAGGGAGTACA*TCAGGTATGTTTAAAGCTGTTCTGTT

Italics is the site of restriction endonuclease or homologous sequence of vector pCCG1/pCCG2 for recombinant plasmid construction using one-step cloning ligation

In this research, tracrRNA and crRNA was fused to a single synthetic guide RNA (sgRNA), which is 82-bp fragment. In order to facilitate the editing procedure, another plasmid pBS-sgRNA was constructed, the procession was as followings: linearized plasmid pBluescript II SK by PCR with primers P19/20, purified the products with SanPrep Column DNA Gel Extraction Kit (Sangon Biotech, China) and digested with *Xba*I-*Nco*I, then ligated it with *Nhe*I-*Nco*I-digested sgRNA (artificial synthesis) using T4 ligase, the products were transformed into *E. coli* DH5α to construct plasmid pBS-sgRNA, which was used as vector for cloning sgRNA fragment.

### Genomic editing procedure

The genomic editing relies on two processes: Cas9 nuclease activity and homologous recombination repair activity. For construction of recombinant plasmid to targeted gene, three elements need to be amplified: sgRNA, up and down homologous sequence flanking the deletion target sequence. In this study, several genes were used as candidates to test this method (Table 3). Take *poxB* deletion as an example, use primers poxBsg-F/sgRNA-R to amplify sgRNA fragment (pBS-sgRNA as template), use primers poxB-U-F/poxB-U-R and poxB-D-F/poxB-D-R to amplify up and down homologous repair arms (*C. glutamicum* genome as template), fuse sgRNA, up and down homologous repair arms to one strand using primers poxB-F/poxB-D-R. N20 sequence was added at the 5’-end of primer poxBsg-F and vector’s homologous sequence at the 5’-end of primer poxB-F, ligate the fused fragment to the *Bam*HI-*Afl*II-digested pCCG1 by the ClonExpress® II One Step Cloning Kit (Vazyme, China), then use chemical transformation to construct the recombinant plasmid pCCG1-*poxB* in *E. coli* DH5α. The sgRNA-homologous arm fragment also can be ligated to pCCG1/pCCG2 with restriction enzyme digestion-T4 ligase method. The design of 20-bp region (N20) with protospacer-adjacent motif (PAM, NGG sequence) matching the targeted gene was programmed artificially or CHOPCHOP/CRISPy-web on net (Blin et al. 2016). In this research, fifteen candidate genes were used to test this strategy, gene knockout: *ldh*, *eutD*, *gabP*, *glnA1*, *glnA2*, *alaT*, *argR*, *gabTD*, *aceAB*, *poxB*; substitution of gene native promoter: *gdhA1*; gene insertion at targeted loci of genome: *eutD*:: *speC*, *gabP*:: *gadB*, *eutD*:: *gdhA, alaT*:: *gdhA*. When insert gene *speC*, *gadB* and *gdhA* into *eutD*, *gabP* and *eutD*, cloning the gene (including promoter PtacM) using primers speC-F/speC-R, gadB-F/gadB-R, gdhA-F/gdhA-R and plasmids pJYW-5-*speC*, pJYW-5-*gadB*, pJYW-5-*gdhA* as template respectively, then ligate the gene between homologous arms to construct the plasmids pCCG1-Δ*eutD*::*speC*, pCCG1-Δ*gabP*::*gadB*, pCCG1-Δ*eutD*::*gdhA*, pCCG1-Δ*alaT*::*gdhA* and pCCG2-Δ*alaT*::*gdhA* in *E. coli* DH5α.

**Table 3:** Gene editing in this study

Strains and Genes	Locus	Plasmids	N20 and PAM sites	LA (bp)^a^
Gene deletion *C. glutamicum* ATCC 13032				
*ldh*	NCgl2810	PCCG1-*ldh*	CAGCATGTAGCGGAATCGAGCGG	916/989
*eutD*	NCgl2657	pCCG1-*eutD*	GTCGCCGTTGTGGACCATCATGG	800/839
*gabP*	NCgl0464	pCCG1-*gabP*	GTGGAGGCATTGATCACCACTGG	553/580
*glnA1*	NCgl2133	pCCG1-*glnA1*	CGTGGAGCATGGTGTTGAAGCGG	700/643
*glnA2*	NCgl2148	pCCG1-*glnA2*	GAAATATCCGCCGTTGTCAGTGG	609/630
*alaT*	NCgl2747	pCCG1-*alaT*	GGTTGCCAGGCTGATGTGCTCGG	739/765
*argR*	NCgl1345	pCCG1-*argR*	TCGCGGGCGATGCTATCTACTGG	564/625
*gabTD*	NCgl0462, NCgl0463	pCCG1-*gabTD*	CCAGTAATCAACCCCAGCGATGG	754/754
*aceAB*	NCgl2248, NCgl2247	pCCG1-*aceAB*	GGAAATCCTCGTACGCCTCTTGG	810/829
*poxB*	NCgl2521	pCCG1-*poxB*	GGTCACCGGATACTTCACCGTGG	644/654
*C. glutamicum* ATCC 14067				
*gabP*	CEY17_02745	pCCG1-*gabP-2*	GTGGAGGCATTGATCACCACTGG	553/580
*C. glutamicum ssp. lactofermentum*				
*gabP*	ND^b^	pCCG1-*gabP-3*	GTGGAGGCATTGATCACCACTGG	553/580
Gene insertion				
Φ(PtacM-*gdhA1*)^c^	NCgl1999	pCCG1-PtacM-*gdhA1*	GCAAACGCCTAGGATGTACATGG	721/662
*eutD*::*speC*	NCgl2657	pCCG1-Δ*eutD*::*speC*	GTCGCCGTTGTGGACCATCATGG	800/839
*gabP*::*gadB*	NCgl0464	pCCG1-Δ*gabP*::*gadB*	GTGGAGGCATTGATCACCACTGG	553/580
*gabP*::*gadB2B1*^*m*^	NCgl0464	pCCG1-Δ*gabP*::*gadB2-gadB1*^*m*^	GTGGAGGCATTGATCACCACTGG	553/580
*eutD*::*gdhA*	NCgl2657	pCCG1-Δ*eutD*::*gdhA*	GTCGCCGTTGTGGACCATCATGG	800/849
*alaT*::*gdhA*	NCgl2747	pCCG1-Δ*alaT*::*gdhA*	GGTTGCCAGGCTGATGTGCTCGG	739/765
*alaT*::*gdhA*	NCgl2747	pCCG2-Δ*alaT*::*gdhA*	GGTTGCCAGGCTGATGTGCTCGG	739/765

a, LA: the length of homologous arms (bp), which is the up and down homologous sequence flanking the deletion target sequence; b, ND: no data; c, Using the strong promoter PtacM and artificial RBS (GAAAGGACTTGAACG) sequence to replace the native promoter of gene *gdhA1* in genome

The recombinant plasmid was purified from *E. coli* DH5α and electroporated into *C. glutamicum*. The electrocompetent of *C. glutamicum* was prepared as reported (Jiang et al. 2017) with modification: inoculate the candidate strain into 4 mL liquid LBHIS media and cultured at 30 °C, 200 rpm for 12–16 h to get pre-culture, 0.5–1 mL of the culture was inoculated into 50 mL media (LBHIS supplemented with Tween-80 1 g/L, l-glycine 25 g/L, l-glycine separately sterilized) and shaking at 30 °C, 200 rpm for 4–5 h until OD_562nm_ reached 0.9–1.0, bath the cells in ice for 15–20 min, centrifugate it at 4000 rpm (~ 3040* g*) for 5–10 min to harvest the cells. Wash the cell twice with pre-cooling 10% (vol. / vol.) glycerol, resuspend it in 1 mL 10% glycerol, split to 80 μL per tube, and store at –70 °C.

For electroporation, ~ 1 μg plasmid was mixed with 80 μL electrocompetent, adding the mixture into pre-cooling 1 mm Gene Pulser cuvette, bath it in ice for 10 min and electroporation at 1.8 kV for twice, the cells were immediately recovered in 0.9 mL pre-cooling liquid LBHIS media for 1–1.5 h in shaker at 30 °C, 150 rpm, then spread the cell on LBHIS agar (supplemented with kanamycin 20 mg/L, isopropyl β-d-1-thiogalactopyranoside, IPTG, 0.01 mM) and culture for 2–3 days at 28 °C until obtain proper size colonies. The colonies were examined by colony PCR method.

### Plasmid curing

Plasmid pCCG1/pCCG2 harbor temperature-sensitive replicon in *C. glutamicum* on account of replicase mutation pBL1^P47S^, when the cultivation temperature higher than 34 °C, the replicase turns to inactivated. For curing the plasmid in positive editing cells, inoculate the cells into 4 mL liquid LBHIS media, shake at 37 °C, 200 rpm for 16 h to obtain proper cell density, plate streaking and cultured at 30 °C for 30–36 h to obtain single colony. Colony PCR or/and kanamycin-resistance LBHIS plates were used to verify plasmid curing, universal primers cas9Test-F (5′-GAAAACCCAATCAACGCATCT-3′)/cas9Test-R (5′-GCTTAGGCAGCACTTTCTCGT-3′) was designed in *cas9* region, which are suitable for all plasmids curing examination, the product is 940-bp-sized fragment if the plasmid existed in cells and nothing for correct curing.

### Gene *gdhA1*, *glnA1* and *glnA2* mutations and l-Glu fermentation

*Corynebacterium glutamicum* is an important industrial strain for l-glutamic acid and l-Glutamine production. Glutamate dehydrogenase (*gdhA1*) and glutamine synthase (*glnA1* and *glnA2*) were key genes related to both amino acids’ biosynthesis in *C. glutamicum* ATCC 13032 (Fig. 2a). In this research, the strong constitutive promoter PtacM and artificial RBS was used to substitute *gdhA1* native promoter in *C. glutamicum* ATCC 13032 chromosome. Amplified targeted sgRNA using primers gdhA1sg-F/sgRNA-R, amplified upstream using primers gdhA1-U-F/gdhA1-U-R, amplified PtacM and downstream using primers gdhA1-D-F/gdhA1-D-R (the plasmid pJYW-5-*gdhA1* as template), fused three fragment by overlap PCR, and ligated the fragment with *Bam*HI-*Afl*II-digested pCCG1 by one-step homologous recombinant cloning kit, transformed the ligated product into *E. coli* DH5α to construct plasmid pCCG1-PtacM-*gdhA1*, and electroporated the purified plasmid into *C. glutamicum* ATCC 13032. The new strain called CGG001. The next, the plasmids pCCG1-*glnA1* and pCCG1-*glnA2* was constructed using similar method to delete gene *glnA1* and *glnA2*, the new strains named as CGN004 and CGN005 respectively. To examine the effect, the strains ATCC 13032, CGG001, CGN004 and CGN005 was cultured in the seed media at 30 °C, 200 rpm for 8–10 h until the OD_562nm_ reached 40 ± 3, inoculate 10% culture in l-glutamic acid fermentation media and fermentation at 30 °C, 200 rpm for 72 h. Supplement urea 4 g/L at 0 h and supplement 2.4 g/L at 10 h, 12 h, 14 h, 17 h and 20 h respectively, the overall additive amount of urea was 16 g/L. The strain CGN004 turns to l-glutamine auxotroph after *glnA1* been deleted, so, proper l-glutamine must be supplemented in media to make the cells survive, 100 mg/L l-glutamine was added in seed media, fermentation broths add 100 mg/L l-glutamine twice at 0 h and 24 h. HPLC (Shi et al. 2013) was used to assay the concentration of l-glutamic acid and l-Glutamine in fermentation broths.

## Results

### Construction of CRISPR/Cas9-assisted system for one-step genomic editing in *C. glutamicum*

**Fig. 1:**
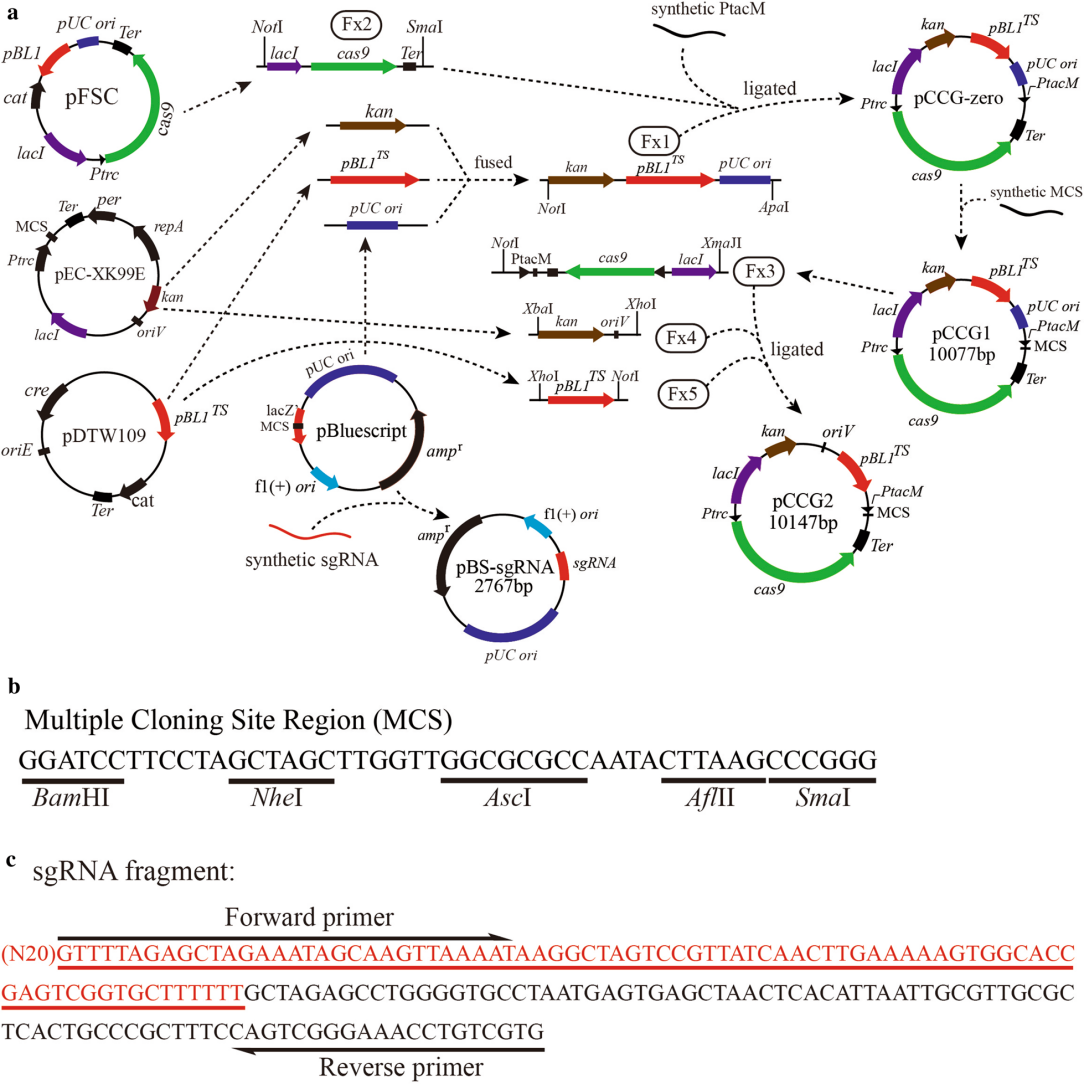
Construction of CRISPR/Cas9-assisted system. **a** The overview diagram of vectors pCCG1, pCCG2 and pBS-sgRNA construction, *E. coli*—*C. glutamicum* shuttle expression vector pCCG1 and pCCG2 is kanamycin resistance, Cas9 nuclease gene is under the control of the inducible promoter P*trc*, pBL1^TS^ is temperature-sensitive replicon in *C. glutamicum*, constitutive promoter PtacM and multiple cloning site (MCS) were artificial synthesis, sgRNA were expressed by promoter PtacM. Small sgRNA sequence was ligated with plasmid pBluescript II SK. sgRNA fragment were amplified from the plasmid pBS-sgRNA when needed. **b** The sequence of multiple cloning sites, including *Bam*HI, *Nhe*I, *Asc* I, *Afl*II and *Sma*I (*Xma*I) restriction enzyme sites. **c** The full nucleotide sequence of sgRNA fragment (171 bp) in plasmid pBS-sgRNA, the red part is Cas9 nuclease recognition site (82 bp)

The *E. coli*—*C. glutamicum* shuttle plasmid of CRISPR/Cas9 vector were constructed and named as pCCG1/pCCG2. The overview diagram of its construction was showed in Fig. 1a. The vector pCCG1 (10077 bp) was first constructed, and then another vector pCCG2 (10147 bp) was following been constructed, both plasmids are functional in genomic editing of *C. glutamicum*. *cas9* expression was designed under the control of inducible promoter *Ptrc*. sgRNA was under constitutive promoter PtacM to insure its expression level. The construction of recombinant plasmid for targeted gene could utilize the method of restriction enzymes digestion-T4 ligase or homologous recombinant cloning kit. The fragment sgRNA, upstream and downstream homologous repair arms could be fused to single strand with overlap PCR technology to promote the construction efficiency of recombinant plasmids in *E. coli*. The sequence of artificial multiple cloning sites (MCS) (Fig. 1b) under PtacM promoter was optimized to ensure every restriction site could be efficiently cut by relevant restriction enzymes. Small sgRNA gene fragment was synthesized and ligated with plasmid pBluescript II SK (the new plasmid named as plasmid pBS-sgRNA, Fig. 1a) to facilitate its conservation and cloning. The full sequence of sgRNA (without N20) in pBS-sgRNA was showed in Fig. 1c.

### Application of the system for genomic editing in *C. glutamicum*

In order to test our CRISPR/cas9 system, gene *ldh*, *eutD*, *gabP*, *glnA1*, *glnA2*, *alaT*, *argR*, *gabTD*, *aceAB*, *poxB* were deleted in *C. glutamicum* ATCC 13032 genome, the results were listed in Table 4. The deletion efficiency of genes was mainly between 26%-90% in *C. glutamicum* ATCC 13032. *gabP* was also deleted in *C. glutamicum* ATCC 14067 and *C. glutamicum* ssp. *lactofermentum* genome, the efficiency was 38.4% and 25.0% respectively, lower than 82.6% in *C. glutamicum* ATCC 13032. Paradoxically, the gene deletion efficiency was great related to gene itself, for example, the deletion efficiency of *argR* in ATCC 13032 was 60.0% using TCGCGGGCGATGCTATCTAC sequence, nevertheless, the gene cluster *argFR* deletion efficiency was 0% when with the same N20 sequence.

**Table 4:** The results of gene editing

Strains and genes	Mutation length (bp)^a^	Results (C/I/T)^b^	Efficiency (%)
Gene deletion *C. glutamicum* ATCC 13032			
* ldh*	568	15/5/20	75.0
* eutD*	877	19/3/22	86.4
* gabP*	930	19/4/23	82.6
* glnA1*	730	13/10/23	56.5
* glnA2*	728	16/7/23	69.6
* alaT*	614	17/6/23	73.9
* argR*	317	6/4/10	60.0
* gabTD*	1949	6/17/23	26.1
* aceAB*	3450	10/13/23	43.5
* poxB*	980	15/8/23	65.2
*C. glutamicum* ATCC 14067			
* gabP*	930	8/15/23	34.8
*C. glutamicum ssp. lactofermentum*			
* gabP*	930	2/6/8	25.0
Gene insertion (deletion/insertion)			
Φ(PtacM-*gdhA1*)	166/150	22/1/23	95.7
* eutD*::*speC*	877/2261	8/15/23	34.8
* gabP*::*gadB*	930/1557	7/16/23	30.4
* gabP*::*gadB2B1*^*m*^	930/3167	5/18/23	21.7
* eutD*::*gdhA*	877/1459	16/7/23	69.6
* alaT*::*gdhA* (pCCG1)	614/1459	6/17/23	26.1
* alaT*::*gdhA* (pCCG2)	614/1459	5/18/23	21.7

a, the mutation length is the length of deleted or/and inserted fragments. 166-bp region has been deleted from genome and 150-bp fragment was inserted when replace native *gdhA1* promoter in *C. glutamicum* ATCC 13032; 877-bp region has been deleted from genome and 1459-bp fragment was inserted when insert *gdhA* into *eutD*; 877-bp region has been deleted from genome and 2261-bp fragment was inserted when insert *speC* into *eutD*; 930-bp region has been deleted from genome and 1557-bp fragment was inserted when insert *gadB* into *gabP*; 930-bp region has been deleted from genome and 3167-bp fragment was inserted when insert gene cluster *gadB2B1*^*m*^ into *gabP*; 614-bp region has been deleted from genome and 1459-bp fragment was inserted when insert *gdhA* into *alaT*. The gene *speC*, *gdhA* and *gadB* are all under the control of PtacM promoter; b, T: the total number of colonies for PCR verification, C: the number of correct colonies, I: the number of incorrect colonies. The efficiency was calculated by (C/T) * 100%

In this research, four genes were inserted into targeted genes in *C. glutamicum* ATCC 13032 genome: *eutD*:: *speC*, *gabP*:: *gadB*, *eutD*:: *gdhA* and *alaT*:: *gdhA*, Based on the experimental results, the efficiency of gene insertion was lower than deletion in most cases, meanwhile, the different gene insertion at same locus might show different efficiency even if using the same N20 and homologous repaired arms. In this research, derived from *E. coli* W3110 gene *speC* (2261 bp, including promoter) and *gdhA* (1459 bp, including promoter) were inserted into *eutD* with same N20 and homologous arms, the efficiency were 34.8% and 69.6% respectively, the editing efficiency was contrary to the size of inserted gene, and lower than *eutD* deletion (86.4%), the efficiency of *gabP*:: *gadB* (30.4%) also lower than *gabP* deletion (82.6%).

The vector pCCG1 and pCCG2 were both used to insert *gdhA* into *alaT*. Gene *gdhA* was cloned from *E. coli* W3110 genome and ligated it between *alaT* up and down homologous arms. The efficiency was 26.1% and 21.7% for pCCG1 and pCCG2 respectively.

### Editing of genes *gdhA1*, *glnA1* and *glnA2* in *C. glutamicum*

**Fig. 2:**
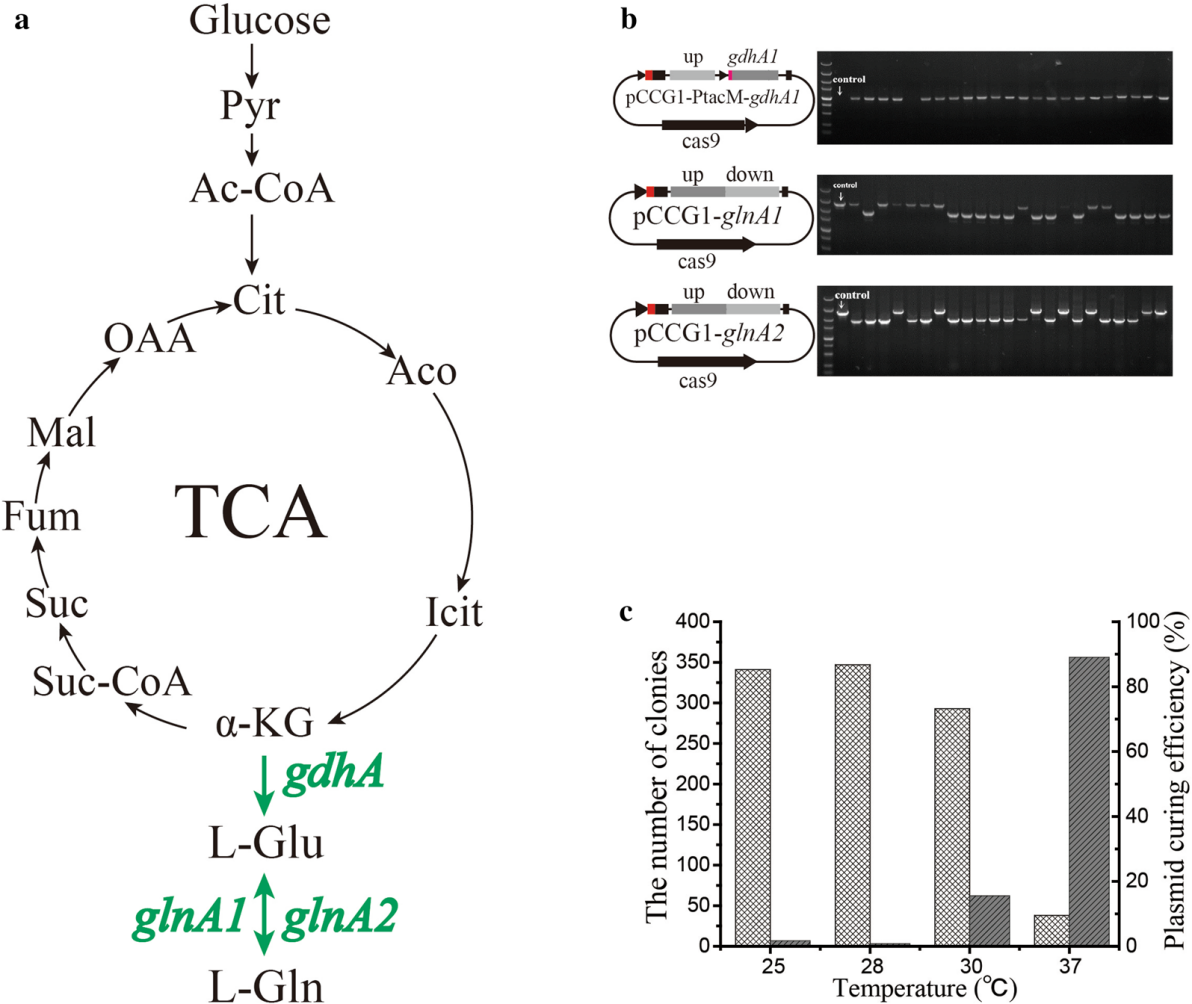
l-Glu and l-Gln biosynthetic pathway and the results of gene editing. **a** l-Glu and l-Gln biosynthetic pathway. Pyr: pyruvate; Ac-CoA: acetyl-CoA; Cit: Citrate; Aco: cis-aconitate; Icit: isocitate; α-KG: alpha-ketoglutaramate; Suc-CoA: succinyl-CoA; Suc: succinate; Fum: fumarate; Mal: malate; OAA: oxaloacetate. **b** The first lane is DNA marker and the second lane is the control before editing, the upstream primer of *gdhA1* was designed in promoter PtacM, there is no PCR product in negative cells. **c** The plasmid stability at different temperature. The cells harboring plasmid were cultured at 25 °C, 28 °C, 30 °C and 37 °C respectively, the total number of colonies were counted by cfu per plate

In this research, the plasmid pCCG1-PtacM-*gdhA1* was designed to replace the *gdhA1* native promoter using the strong promoter PtacM and artificial RBS, the plasmid profile and agarose gel eletrophoresis was showed in Fig. 2b, pCCG1-*glnA1* and pCCG1-*glnA2* were used to delete *glnA1* and *glnA2* from chromosome. The efficiency was 95.7%, 56.5% and 69.6% respectively.

In this research, the plasmid pCCG1-PtacM-*gdhA1* was used to study the stability of recombinant plasmid pCCG1/pCCG2 in *C. glutamicum*. Some cells which harboring plasmid pCCG1-PtacM-*gdhA1* was cultured at different temperature, the statistical result was showed in Fig. 2c. The cells showed maximum stability when cultured at 28 °C, while the plasmid will start to loss when the temperature higher than 30 °C, the plasmid curing reached nearly 90% at 37 °C, the stability at 25 °C was similar to 28 °C while the cell growth rate will decrease and editing cycle increase at least one day, so the 28 °C is optimal culture temperature when the cells harboring recombinant plasmid pCCG1/pCCG2.

### Comparison of l-Glu and l-Gln production in the mutant strains

**Fig. 3:**
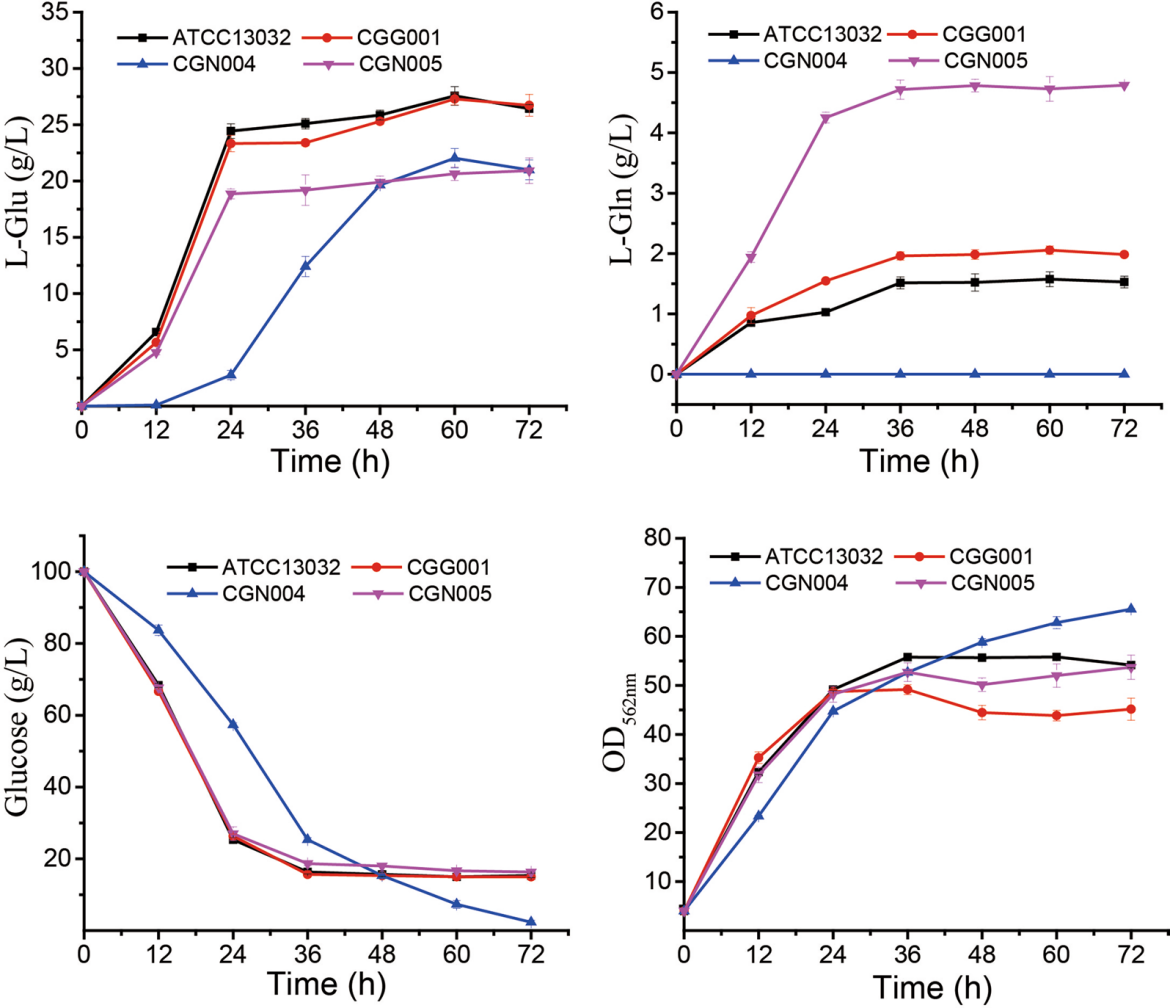
The fermentation results of the strains CGG001, CGN004 and CGN005. *C. glutamicum* ATCC 13032 was used as control. l-Glu: l-Glutamic acid; l-Gln: l-Glutamine. The results showed the concentration of l-Glu and l-Gln in fermentation broths. Meanwhile, the cell density (OD_562nm_) and the glucose concentration also been showed in the results. Data was shown as mean ± CI (Confidence Interval), 3 independent experiments for statistics, α = 0.01

The new strains CGG001, CGN004, CGN005 and ATCC 13032 (as control) were cultured in l-Glutamic acid fermentation media, the results were depicted in the Fig. 3. The concentration of the l-Glutamic acid reached maximum at 60 h, the strains CGN004 (22.03 g/L) and CGN005 (20.64 g/L) decreased 20.1% and 25.1% compared to ATCC 13032 (27.57 g/L). The l-glutamine concentration in CGG001 (2.06 g/L) and CGN005 (4.73 g/L) broths increased 30.4% and 199.4% compared to ATCC 13032 (1.58 g/L), there is no l-glutamine could be detected in CGN004 broths. The cell density of ATCC 13032, CGG001 and CGN005 reached maximum at 36 h while CGN004 was increasing in 72 h and 1.21-fold than ATCC 13032. The average glucose consumption rate decreased 42.9% in CGN004 compared to ATCC 13032 in 24 h.

## Discussion

**Fig. 4:**
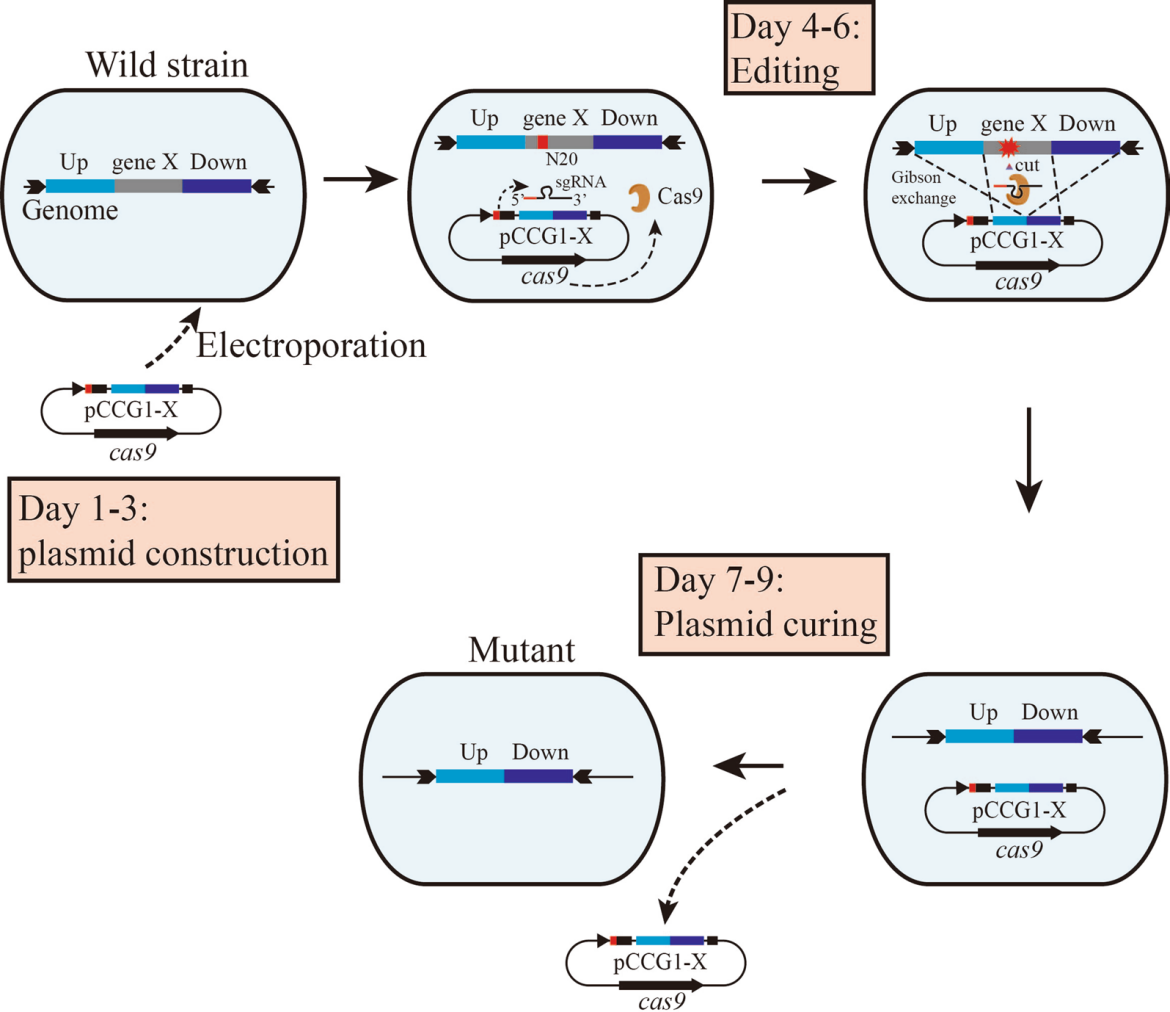
The diagram of genomic editing procedure. **a** Day 1–3: The recombinant plasmid construction for targeted gene X in *E. coli* DH5α. Day 4–6, electroporate the plasmid into the electrocompetent cell of *C. glutamicum* and cultured for 2–3 days at 28 °C, examine the colonies using colony PCR method. N20 sequence at 5′-end of sgRNA was complementary to the targeted gene X, which guide Cas9 to break genome at PAM (NGG) site, the upstream and downstream homologous sequence in plasmid was used to repair the DSBs. Day 7–9, cure the plasmids in positive cells. If continuous editing was needed, make electrocompetent cells of the positive cells, electroporate another plasmid into it for the next editing cycle

In this research, an improved method of CRISPR/Cas9-assisted system for *C. glutamicum* was established, all elements for the gene editing were designed in one vector pCCG1/pCCG2. Gene editing could be done by electrotrasformation at one time. the recombinant plasmid for targeted gene could be one-step constructed in *E. coli* DH5α, the overall procedure of genomic editing was showed in Fig. 4, this method demonstrates simplest operation and time-saving compared to the reported, 8–9 days can complete the whole work of genomic editing and 5 days for continuous editing (the next plasmid was prepared before electrotransformation), so this method is highly efficient and suitable for abundant gene mutation in *C. glutamicum*.

*Corynebacterium glutamicum* is an important industrial microorganism, the studies of metabolic engineering were due to rationally design and optimize the metabolic pathway, these strategies’ implementation often based on high efficient genomic editing tools. The reported genomic editing methods were mainly multiple-plasmid system (Peng et al. 2017; Zhao et al. 2020), or integrated *cas9*/*recET* into chromosome before editing targeted gene with traditional method (Cho et al. 2017), the processes of plasmid construction, introduced one by one, and plasmid curing were complex. Wang et al. (Wang et al. 2018) introduced *cas9* and *recET* into chromosome using plasmids pIN-*cas9* and pIN-*recET* in advance, the plasmids were SacB-based method and need two rounds of homologous recombination, so these methods are labor-intensive and gene *cas9*/*recET* remain on chromosome after editing, sometimes, the exogenous *cas9*/*recET* in chromosome might disturbed certain metabolism of *C. glutamicum* and not suitable to the studies of metabolic engineering. Our method designed *cas9* in plasmid and don’t need introduce any genes or plasmid into chromosome before genomic editing, meanwhile, there was no residual sequence in chromosome after editing, this is another advantage for metabolic engineering research in *C. glutamicum*. Peng (Peng et al. 2018) constructed two-plasmid system and improved it all-in-one CRISPR/Cas9 system, the chloramphenicol resistant pFSTC is 11.2-kb-sized vector, larger than pCCG1/pCCG2 (10 kb), so pCCG1/pCCG2 have larger loading capacity than pFSTC theoretically. Furthermore, *C. glutamicum* had higher resistance to kanamycin than chloramphenicol, the incubation time of *C. glutamicum* harboring pCCG1/pCCG2 on LBHIS plate will decrease 2–3 days than pFSTC, pCCG1/pCCG2 indicated shorter editing cycle and more efficient.

The genomic editing of *C. glutamicum* was time-consuming and low efficiency, the efficiency of Cas9/Cpf1-based editing were often between 20–90%, The plasmid pCCG1/pCCG2 also have some deficiencies as well, it’s hard to reach 100% might because the lack of typical CRISPR RNA sequence in *C. glutamicum* (Jiang et al. 2017) or Off-Target effect of Cas9 nuclease (Fu et al. 2013; Shen et al. 2014, 2019). In this research, take *poxB* deletion in ATCC 13032 as example, N20 sequence of AATCAGCAGATCCGCCTCAT cannot target Cas9 to cut off the genome but GGTCACCGGATACTTCACCG sequence did. Large fragments or genes (> 3 kb) insertion were not suggested due to the size of plasmids, the vector pCCG1/pCCG2 were about 10 kb, the editing efficiency will dramatically decline when the recombinant plasmid larger than 14 kb, 1.6-kb and 3.2-kb fragment integration into *gabP*, the efficiency was 30.4% and 21.7% respectively (Table 4). So large gene (> 3.5 kb) integration in chromosome was suggested to break the gene into some fragments and insert it one by one. Both of pCCG1 and pCCG2 were effective in *C. glutamicum* while there were some differences, pCCG1 might have higher efficiency while pCCG2 have higher concentration extract from *E. coli* DH5α, pCCG1 was mainly used in this research. To compare the pCCG1 and pCCG2, *gdhA* was inserted into *alaT* by pCCG1 and pCCG2 with same N20 and repair arms, the efficiency is 26.1% and 21.7% respectively (Table 4), we lack enough data to compare the efficiency of pCCG1 and pCCG2, but both of them were effective in *C. glutamicum*.

This method was ideal in *C. glutamicum* ATCC 13032 and ATCC 14067, other strains of *C. glutamicum* might show lower effective (Table 4), perhaps the replicon pBL1^TS^ was not suitable for these strains. Some recombinant plasmids might impact the growth of *E. coli* DH5α, the cells’ growth rate and density might lower than regular *E. coli* DH5α harboring empty vector pCCG1/pCCG2. To solve this problem, *E. coli* DH5α could be cultured for longer time (16–20 h) or use not less than 5 mL culture to extract recombinant plasmids.

Cas9 nuclease is toxic for *C. glutamicum*. In this strategy, *cas9* were expressed under inducible promoter P*trc*, low concentration IPTG were supplemented in media to limit *cas9* expression, 0.01–0.05 mM IPTG was proper concentration and can make the cell survived (0.01 mM was suggested). Cas9 limited-expression can be achieved with two methods: first, supplement 0.01–0.02 mM IPTG in LBHIS agar plates, this method can make *cas9* little continuous expression; second, supplement 0.1 mM IPTG in liquid recovery LBHIS media after electroporation and short-time induce *cas9* expression for 1–1.5 h, then centrifugate the culture to collect the cell, discard the supernatant to remove IPTG, resuspend the cells in 0.1 mL LBHIS and plate streaking, the first method maybe more convenient. sgRNA fragment was amplified from plasmid pBS-sgRNA, because sgRNA is 82-bp-sized small fragment, we amplified downwards the sequence to obtain about 200-bp-size production (Fig. 1c) to facilitate the later experiment (sgRNA-R was used as universal primer). This CRISPR/Cas9-assisted method needs homology-directed system to repair double-strand breaks (DSBs) (Malyarchuk et al. 2007; Wilson et al. 2003), the proper length of homologous repaired arms were suggested between 500–1000 bp. pCCG1/pCCG2 is temperature-sensitive plasmid in *C. glutamicum* due to the replicon pBL1^P47S^ mutation, plasmid pCCG1-PtacM-*gdhA1* was used to test its stability in *C. glutamicum* ATCC 13032 (Fig. 2c), the colonies in plates were counted at 25 °C, 28 °C, 30 °C and 37 °C. It’s stable at 25–28 °C and will lost when the temperature ≥ 30 °C. The plasmid curing efficiency will up to 90% at 37 °C. It showed the maximum number of colonies at 28 °C, so 28 °C was utilized to culture the cells harboring recombinant plasmid pCCG1/pCCG2 considering with Cas9 nuclease activity, homologous repaired activity and cell growth velocity. Meanwhile, the incubation time could be decreased one day at 28 °C compared to 25 °C on the LBHIS plates, and revealed higher efficiency for large gene editing (> 2 kb). The cells harboring recombinant plasmid pCCG1/pCCG2 sometimes appeared some extremely big colonies on LBHIS plates after electrotansformation, these colonies were fast-growing (might appeared in one day on plate) compared to regular one on LBHIS plates, it is not correct mutant and not necessary to examine it with colony PCR method, the reasons of this phenomenon are unknown, perhaps related to the replicon itself.

## Data Availability

The datasets generated and/or analyzed during the current study are available from the corresponding author on reasonable request.

## Abbreviations

*E. coli*: *Escherichia coli*
PCR: Polymerase chain reaction
IPTG: Isopropyl β-d-1-thiogalactopyranoside
LBHIS: LB media containing brain heart infusion solution

## References

[CR1] Altenbuchner J (2016). Editing of the *Bacillus subtilis* genome by the CRISPR–Cas9 system. Appl Environ Microbiol.

[CR2] Austin S, Ziese M, Sternberg N (1981). A novel role for site-specific recombination in maintenance of bacterial replicons. Cell.

[CR3] Blin K, Pedersen LE, Weber T, Lee SY (2016). CRISPy-web: an online resource to design sgRNAs for CRISPR applications. Synth Syst Biotechnol.

[CR4] Braga A, Oliveira J, Silva R, Ferreira P, Rocha I, Kallscheuer N, Marienhagen J, Faria N (2018). Impact of the cultivation strategy on resveratrol production from glucose in engineered *Corynebacterium glutamicum*. J Biotechnol.

[CR5] Cameron Coates R, Blaskowski S, Szyjka S, van Rossum HM, Vallandingham J, Patel K, Serber Z, Dean J (2019). Systematic investigation of CRISPR–Cas9 configurations for flexible and efficient genome editing in *Corynebacterium glutamicum* NRRL-B11474. J Ind Microbiol Biotechnol.

[CR6] Chen X, Gao C, Guo L, Hu G, Luo Q, Liu J, Nielsen J, Chen J, Liu L (2017). DCEO biotechnology: tools to design, construct, evaluate, and optimize the metabolic pathway for biosynthesis of chemicals. Chem Rev.

[CR7] Cheng F, Yu H, Stephanopoulos G (2019). Engineering *Corynebacterium glutamicum* for high-titer biosynthesis of hyaluronic acid. Metab Eng.

[CR8] Cho JS, Choi KR, Prabowo CPS, Shin JH, Yang D, Jang J, Lee SY (2017). CRISPR/Cas9-coupled recombineering for metabolic engineering of *Corynebacterium glutamicum*. Metab Eng.

[CR9] Cleto S, Jensen JVK, Wendisch VF, Lu TK (2016). *Corynebacterium glutamicum* Metabolic Engineering with CRISPR Interference (CRISPRi). ACS Synth Biol.

[CR10] Fu Y, Foden JA, Khayter C, Maeder ML, Reyon D, Joung JK, Sander JD (2013). High-frequency off-target mutagenesis induced by CRISPR-Cas nucleases in human cells. Nat Biotechnol.

[CR11] Fukui K, Nanatani K, Nakayama M, Hara Y, Tokura M, Abe K (2019). *Corynebacterium glutamicum* CgynfM encodes a dicarboxylate transporter applicable to succinate production. J Biosci Bioeng.

[CR12] Gauttam R, Seibold GM, Mueller P, Weil T, Weiß T, Handrick R, Eikmanns BJ (2019). A simple dual-inducible CRISPR interference system for multiple gene targeting in *Corynebacterium glutamicum*. Plasmid.

[CR13] Hu J, Tan Y, Li Y, Hu X, Xu D, Wang X (2013). Construction and application of an efficient multiple-gene-deletion system in *Corynebacterium glutamicum*. Plasmid.

[CR14] Hu J, Li Y, Zhang H, Tan Y, Wang X (2014). Construction of a novel expression system for use in *Corynebacterium glutamicum*. Plasmid.

[CR15] Jager W, Schafer A, Puhler A, Labes G, Wohlleben W (1992). Expression of the *Bacillus subtilis sacB* gene leads to sucrose sensitivity in the gram-positive bacterium *Corynebactenium glutamicum* but not in *Streptomyces lividans*. J Bacteriol.

[CR16] Jiang Y, Chen B, Duan C, Sun B, Yang J, Yang S (2015). Multigene editing in the *Escherichia coli* genome via the CRISPR-Cas9 system. Appl Environ Microbiol.

[CR17] Jiang Y, Qian F, Yang J, Liu Y, Dong F, Xu C, Sun B, Chen B, Xu X, Li Y, Wang R, Yang S (2017). CRISPR-Cpf1 assisted genome editing of *Corynebacterium glutamicum*. Nat Commun.

[CR18] Liu X, Yang Y, Zhang W, Sun Y, Peng F, Jeffrey L, Harvey L, McNeil B, Bai Z (2016). Expression of recombinant protein using *Corynebacterium Glutamicum*: progress, challenges and applications. Crit Rev Biotechnol.

[CR19] Liu J, Wang Y, Lu Y, Zheng P, Sun J, Ma Y (2017). Development of a CRISPR/Cas9 genome editing toolbox for *Corynebacterium glutamicum*. Microb Cell Fact.

[CR20] Lv Y, Wu Z, Han S, Lin Y, Zheng S (2012). Construction of recombinant *Corynebacterium glutamicum* for L-threonine production. Biotechnol Bioproc E.

[CR21] Malyarchuk S, Wright D, Castore R, Klepper E, Weiss B, Doherty AJ, Harrison L (2007). Expression of *Mycobacterium tuberculosis* Ku and Ligase D in *Escherichia coli* results in RecA and RecB-independent DNA end-joining at regions of microhomology. DNA Repair (amst).

[CR22] Mindt M, Heuser M, Wendisch VF (2019). Xylose as preferred substrate for sarcosine production by recombinant *Corynebacterium glutamicum*. Bioresour Technol.

[CR23] Mitsui R, Yamada R, Ogino H (2019). CRISPR system in the yeast *Saccharomyces cerevisiae* and its application in the bioproduction of useful chemicals. World J Microbiol Biotechnol.

[CR24] Nodvig CS, Hoof JB, Kogle ME, Jarczynska ZD, Lehmbeck J, Klitgaard DK, Mortensen UH (2018). Efficient oligo nucleotide mediated CRISPR-Cas9 gene editing in *Aspergilli*. Fungal Genet Biol.

[CR25] Oh JH, van Pijkeren JP (2014). CRISPR-Cas9-assisted recombineering in *Lactobacillus reuteri*. Nucleic Acids Res.

[CR26] Peng F, Wang X, Sun Y, Dong G, Yang Y, Liu X, Bai Z (2017). Efficient gene editing in *Corynebacterium glutamicum* using the CRISPR/Cas9 system. Microb Cell Fact.

[CR27] Peng F, Liu X, Wang X, Chen J, Liu M, Yang Y, Bai Z (2018). Triple deletion of clpC, porB, and mepA enhances production of small ubiquitin-like modifier-N-terminal pro-brain natriuretic peptide in *Corynebacterium glutamicum*. J Ind Microbiol Biotechnol.

[CR28] Pyne ME, Moo-Young M, Chung DA, Chou CP, Kivisaar M (2015). Coupling the CRISPR/Cas9 system with lambda red recombineering enables simplified chromosomal gene replacement in *Escherichia coli*. Appl Environ Microbiol.

[CR29] Sasaki Y, Eng T, Herbert RA, Trinh J, Chen Y, Rodriguez A, Gladden J, Simmons BA, Petzold CJ, Mukhopadhyay A (2019). Engineering *Corynebacterium glutamicum* to produce the biogasoline isopentenol from plant biomass hydrolysates. Biotechnol Biofuels.

[CR30] Schwentner A, Feith A, Munch E (2018). Metabolic engineering to guide evolution – Creating a novel mode for L-valine production with *Corynebacterium glutamicum*. Metab Eng.

[CR31] Shen B, Zhang W, Zhang J, Zhou J, Wang J, Chen L, Wang L, Hodgkins A, Iyer V, Huang X, Skarnes WC (2014). Efficient genome modification by CRISPR–Cas9 nickase with minimal off-target effects. Nat Methods.

[CR32] Shen CC, Hsu MN, Chang CW, Lin MW, Hwu JR, Tu Y, Hu YC (2019). Synthetic switch to minimize CRISPR off-target effects by self-restricting Cas9 transcription and translation. Nucleic Acids Res.

[CR33] Shi F, Li K, Huan X, Wang X (2013). Expression of NAD(H) kinase and glucose-6-phosphate dehydrogenase improve NADPH supply and L-isoleucine biosynthesis in *Corynebacterium glutamicum* ssp. lactofermentum. Appl Biochem Biotechnol.

[CR34] Shi F, Luan M, Li Y (2018). Ribosomal binding site sequences and promoters for expressing glutamate decarboxylase and producing gamma-aminobutyrate in *Corynebacterium glutamicum*. AMB Express.

[CR35] Shi F, Zhang S, Li Y, Lu Z (2019). Enhancement of substrate supply and ido expression to improve 4-hydroxyisoleucine production in recombinant *Corynebacterium glutamicum* ssp. lactofermentum. Appl Microbiol Biotechnol.

[CR36] Song X, Huang H, Xiong Z, Ai L, Yang S (2017) CRISPR-Cas9(D10A) Nickase-Assisted Genome Editing in *Lactobacillus casei*. Appl Environ Microbiol 83(22) 10.1128/AEM.01259-17 10.1128/AEM.01259-17 PMC5666132 28864652

[CR37] Suzuki N, Tsuge Y, Inui M, Yukawa H (2004). Cre/loxP-mediated deletion system for large genome rearrangements in *Corynebacterium glutamicum*. Appl Microbiol Biotechnol.

[CR38] Tan Y, Xu D, Li Y, Wang X (2012). Construction of a novel sacB-based system for marker-free gene deletion in *Corynebacterium glutamicum*. Plasmid.

[CR39] Wada M, Sawada K, Ogura K, Shimono Y, Hagiwara T, Sugimoto M, Onuki A, Yokota A (2016). Effects of phosphoenolpyruvate carboxylase desensitization on glutamic acid production in *Corynebacterium glutamicum* ATCC 13032. J Biosci Bioeng.

[CR40] Wang X (2019). Strategy for improving L-isoleucine production efficiency in *Corynebacterium glutamicum*. Appl Microbiol Biotechnol.

[CR41] Wang B, Hu Q, Zhang Y, Shi R, Chai X, Liu Z, Shang X, Zhang Y, Wen T (2018). A RecET-assisted CRISPR-Cas9 genome editing in *Corynebacterium glutamicum*. Microb Cell Fact.

[CR42] Wang Y-Y, Xua J-Z, Zhanga W-G (2019). Metabolic engineering of l-leucine production in *Escherichia coli* and *Corynebacterium glutamicum*: a review. Crit Rev Biotechnol.

[CR43] Wei L, Xu N, Wang Y, Zhou W, Han G, Ma Y, Liu J (2018). Promoter library-based module combination (PLMC) technology for optimization of threonine biosynthesis in *Corynebacterium glutamicum*. Appl Microbiol Biotechnol.

[CR44] Wen J, Bao J (2019). Engineering *Corynebacterium glutamicum* triggers glutamic acid accumulation in biotin-rich corn stover hydrolysate. Biotechnol Biofuels.

[CR45] Westbrook AW, Moo-Young M, Chou CP (2016). Development of a CRISPR–Cas9 tool kit for comprehensive engineering of *Bacillus subtilis*. Appl Environ Microbiol.

[CR46] Wilson TE, Topper LM, Palmbos PL (2003). Non-homologous end-joining: bacteria join the chromosome breakdance. Trends Biochem Sci.

[CR47] Xiao J, Wang D, Wang L, Jiang Y, Xue L, Sui S, Wang J, Guo C, Wang R, Wang J, Li N, Fan H, Lv M (2020). Increasing l-lysine production in *Corynebacterium glutamicum* by engineering amino acid transporters. Amino Acids.

[CR48] Yang Z, Pei X, Xu G, Wu J, Yang L (2019). Efficient inducible expression of nitrile hydratase in *Corynebacterium glutamicum*. Process Biochem.

[CR49] Zha J, Zang Y, Mattozzi M, Plassmeier J, Gupta M, Wu X, Clarkson S, Koffas MAG (2018). Metabolic engineering of *Corynebacterium glutamicum* for anthocyanin production. Microb Cell Fact.

[CR50] Zhang C, Meng X, Wei X, Lu L (2016). Highly efficient CRISPR mutagenesis by microhomology-mediated end joining in *Aspergillus fumigatus*. Fungal Genet Biol.

[CR51] Zhang Y, Wang J, Wang Z, Zhang Y, Shi S, Nielsen J, Liu Z (2019). A gRNA-tRNA array for CRISPR-Cas9 based rapid multiplexed genome editing in *Saccharomyces cerevisiae*. Nat Commun.

[CR52] Zhang Y, Zhang X, Xiao S, Qi W, Xu J, Yuan Z, Wang Z (2019). Engineering *Corynebacterium glutamicum* Mutants for 3-Methyl-1-butanol Production. Biochem Genet.

[CR53] Zhao N, Li L, Luo G, Xie S, Lin Y, Han S, Huang Y, Zheng S (2020). Multiplex gene editing and large DNA fragment deletion by the CRISPR/Cpf1-RecE/T system in *Corynebacterium glutamicum*. J Ind Microbiol Biotechnol.

